# Diagnosing Paget-Schroetter Syndrome Using Point of Care Ultrasound (POCUS)

**DOI:** 10.24908/pocus.v7i1.15364

**Published:** 2022-04-21

**Authors:** Khaled Taha, Tomás Breslin, John M Moriarty, Shammy Ali, Bernhard Louw

**Affiliations:** 1 Accident and Emergency Department, The Mater Misericordiae University Hospital Dublin Ireland; 2 Department of Interventional Radiology, The Mater Misericordiae University Hospital Dublin Ireland

**Keywords:** POCUS, Paget-Schroetter Syndrome, subclavian vein thrombosis

## Abstract

Paget-Schroetter Syndrome, or effort thrombosis, is a relatively rare disorder. It refers to axillary-subclavian vein thrombosis (ASVT) that is associated with strenuous and repetitive activity of the upper extremities 1. Anatomical abnormalities at the thoracic outlet and repetitive trauma to the endothelium of the subclavian vein are key factors in its initiation and progression. Doppler ultrasonography is the preferred initial test, but contrast venography is the gold standard for diagnosis 1, 2. Early diagnosis coupled with a multimodal treatment strategy is crucial for optimal outcomes. We present a case of a 21-year-old male in which point of care ultrasound (POCUS) expedited the diagnosis and subsequent early treatment of right subclavian vein thrombosis. He presented to our Emergency Department with acute swelling, pain and erythema of his right upper limb. He was promptly diagnosed to have thrombotic occlusion of the right subclavian vein using POCUS in our Emergency Department.

## Case Report

A 21-year-old male who is an avid sportsman presented to the emergency department with a 3-day history of pain and swelling of the right upper limb. He had joined a gym club three months prior where he partakes in weightlifting. His only past medical history was COVID-19 12 months prior. He had no family history of thromboembolic disease. 

His examination revealed a swollen and erythematous right upper limb. His peripheral pulses were palpable and comparable to the opposite side. His range of movements were intact. The left upper limb and both lower limbs were normal. He had no symptoms of pulmonary embolism. 

**Figure 1:**
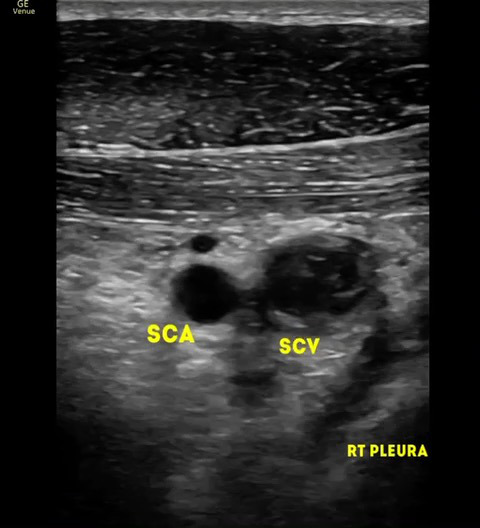
Transverse view with linear probe over the right medial end of the clavicle shows the right subclavian vein being noncompressible with an echogenic thrombus inside.

**Figure 2:**
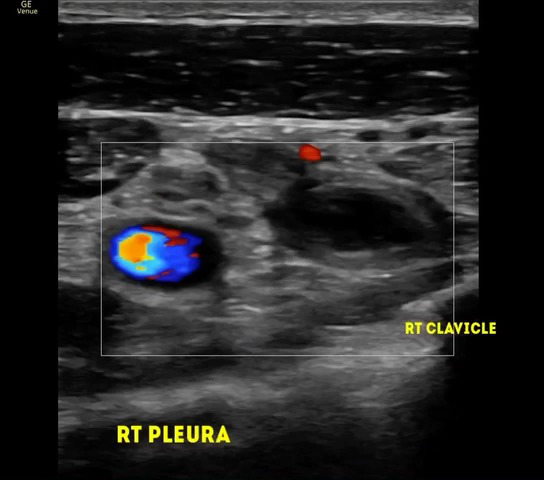
Transverse view with linear probe over the right medial end of the clavicle shows goodcolour flow within the right subclavian artery but the right subclavian vein lying above the medial end of the right clavicle shows no colour flow with same echogenic thrombus inside.

**Figure 3:**
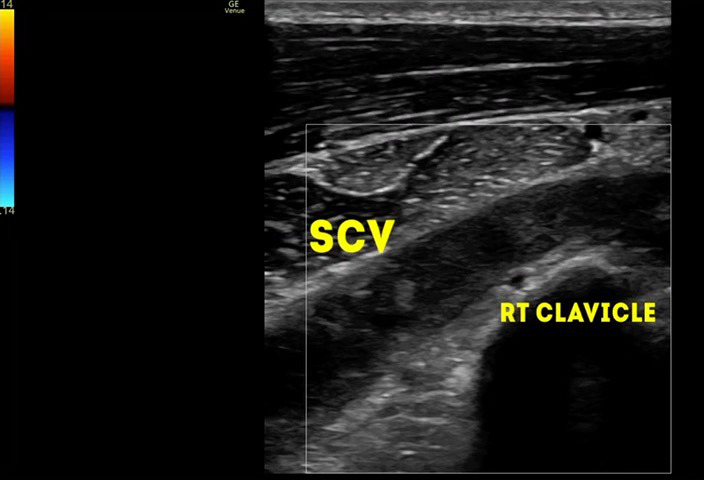
In long axis view with linear probe over the right medial end of the clavicle, POCUScolour doppler demonstrates the right subclavian vein above the right clavicle which is non- compressible.

**Figure 4:**
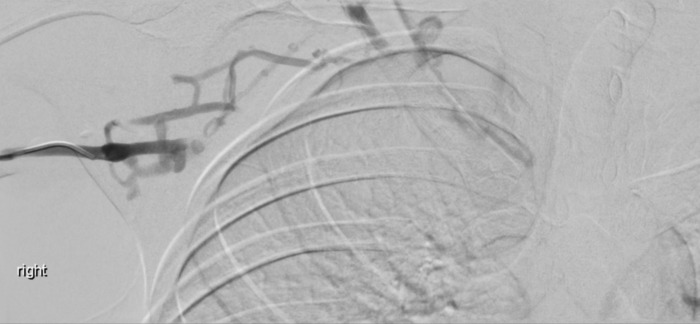
Digital subtraction venogram from the right arm demonstrates thrombotic occlusion of the right subclavian vein with collateralization.

**Figure 5:**
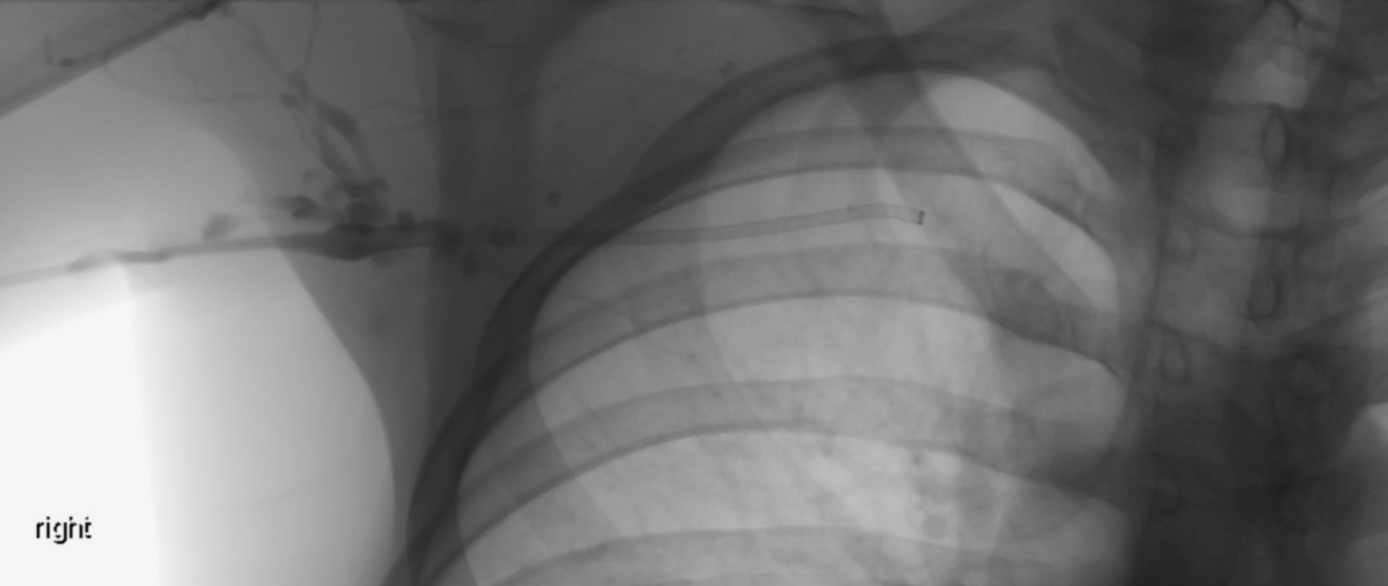
Aspiration thrombectomy performed with Penumbra Indigo Cat 8 device.

**Figure 6:**
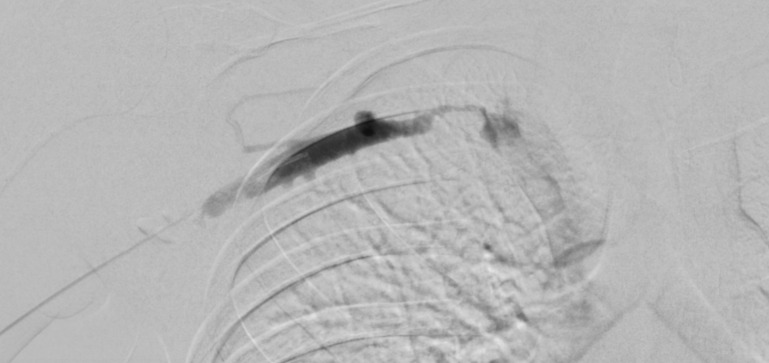
Completion venography demonstrating high grade stenosis of the right subclavian vein with due to compression by the clavicle and first rib, consistent with Thoracic Outlet Syndrome (TOS). No residual thrombus is identified.

Right upper limb POCUS was concerning for subclavian vein thrombus with a distended right subclavian vein. The findings are demonstrated in Figures 1-3 and corresponding Videos S1-S3. Blood work up revealed a non-negative D-dimer at 0.93 mg/l (reference range <0.50mg/l). His chest x-ray showed no obvious abnormalities, and no evidence of an accessory rib. Once the diagnosis of subclavian vein thrombosis was made, low-molecular-weight heparin was initiated, and he was admitted under General Medicine. An urgent formal ultrasound confirmed an acute 6 cm thrombus within the right subclavian vein . Furthermore, it showed normal compressibility and colour flow within the right basilic vein, brachial vein, cephalic vein and axillary vein without any evidence of thrombosis. The patient underwent thrombectomy and venoplasty by Interventional Radiology the next day due to severity of symptoms, followed with anticoagulation. These are demonstrated in Figures 4-6. Video S4 shows the initial digital subtraction venogram from the right arm which demonstrates thrombotic occlusion of the right subclavian vein with collateralization. 

He was initially treated with Low Molecular Weight Heparin (LMWH), then switched to Apixaban. The IR venogram showed an acute thrombotic occlusion of the right subclavian vein with a high-grade stenosis at the junction of the first rib and clavicle. The findings were consistent with Paget-Schroetter Syndrome, with venous thoracic outlet obstruction. He was discharged on anticoagulation and is being followed up for consideration of a first rib resection. The importance of POCUS in this case is demonstrated through the 1-day turnaround between diagnosis and interventional treatment.

## Discussion

Paget-Schroetter Syndrome, or effort thrombosis, usually follows vigorous sporting activities, such as wrestling, playing ball, gymnastics and swimming, which involve upper extremity movements. Hyperabduction and extension of the arm involved with these activities cause undue strain on the subclavian vein leading to micro trauma of the endothelium and activation of the coagulation cascade. Paget-Schroetter Syndrome is categorized as a venous variant of thoracic outlet syndrome and accounts for 30–40% of spontaneous axillary-subclavian vein thrombosis (ASVT) and for 10–20% of all upper extremity deep venous thrombosis (UEDVT) 2, 3, 4. Patients can present in different ways, ranging from asymptomatic to acute, intense pain and swelling. POCUS has been instrumental in diagnosing cases promptly in the emergency department. Emergency physicians using POCUS as initial line of investigation is important for the early diagnosis and treatment of this disorder. CT Venogram can be used in ultrasound negative cases which have high index of suspicion. 

Common complications of Paget-Schroetter syndrome are pulmonary embolism and post thrombotic syndrome 2. PE due to Paget-Schroetter Syndrome is now thought to have an incidence of 10–25% 5. Post-thrombotic syndrome has a high morbidity associated with chronic pain, swelling, discoloration, edema, ulcers and varicose vein formation. Its incidence varies between 7% and 46% 5. Management is with anticoagulation, systemic thrombolysis, or catheter directed thrombolysis. A survey conducted in UK regarding the most favored approaches to treat Paget-Schroetter Syndrome revealed that most surgeons favored a combined interventional radiology and surgical approach; 17% favored conservative management and 86.7% favored thrombolysis followed by elective thoracic outlet decompression procedure; 65% did not favor stenting. First rib resection was the most favored surgical procedure (74%) and trans-axillary approach was favored by majority (55%) 3. Importance of prompt diagnosis within the emergency department is crucial to prevent these complications and emergency physicians must be vigilant to not miss such rare cases of upper limb thrombosis.

## Conflict of interest

JMM has professional links to Angiodynamics, Boston Scientific, Penumbra, Medtronic, Retriever, Pavmed, Inquis, Innova Vascular. The other authors have no disclosures to declare.

## Funding

The authors received no funding.

## Patient consent

The authors gained consent from the patient to publish. 

## Supplementary Material

 Video S1 Transverse view with linear probe over the right medial end of the clavicle shows the right subclavian vein being noncompressible with an echogenic thrombus inside.

Video S2 Transverse view with linear probe over the right medial end of the clavicle shows good colour flow within the right subclavian artery but the right subclavian vein lying above the medial end of the right clavicle shows no colour flow with same echogenic thrombus inside.

Video S3 In long axis view with linear probe over the right medial end of the clavicle, POCUS colour doppler demonstrates the right subclavian vein above the right clavicle which is non- compressible.

Video S4 Digital subtraction venogram from the right arm demonstrates thrombotic occlusion of the right subclavian vein with collateralization.
